# Supplementary material for: Tumor-Targeting Peptides: The Functional Screen of Glioblastoma Homing Peptides to the Target Protein FABP3 (MDGI)
Source: Cancers (Basel). 2020 Jul 8;12(7):1836. doi: 10.3390/cancers12071836 (PMC7409020; doi:10.3390/cancers12071836)
Supplement: Supplementary file 1 [file cancers-12-01836-s001.zip › cancers-835234-supplementary-resubmit - layout.pdf]

# Tumor-Targeting Peptides: The Functional Screen of Glioblastoma Homing Peptides to the Target Protein FABP3 (MDGI)

**Abiodun Ayo [1], Eduard Figueras [2], Thomas Schachtsiek [2], Mazlum Budak [2], Norbert Sewald [2] and Pirjo Laakkonen [1,3,*]**

[1] Translational Cancer Medicine Research Program, Faculty of Medicine, University of Helsinki, 00014 Helsinki, Finland; abiodun.ayo@helsinki.fi

[2] Organic and Bioorganic Chemistry OC III, Department of Chemistry, Bielefeld University, 33615 Bielefeld, Germany; figueraseduard@gmail.com (E.F.); T.Schachtsiek@uni-bielefeld.de (T.S.); mazlum.budak@uni-bielefeld.de (M.B.); norbert.sewald@uni-bielefeld.de (N.S.)

[3] Laboratory Animal Center, Helsinki Institute of Life Science (HiLIFE), University of Helsinki, 00014 Helsinki, Finland

* Correspondence: pirjo.laakkonen@helsinki.fi; Tel.: +358-50-4489100

**Supplementary Figures and Tables**

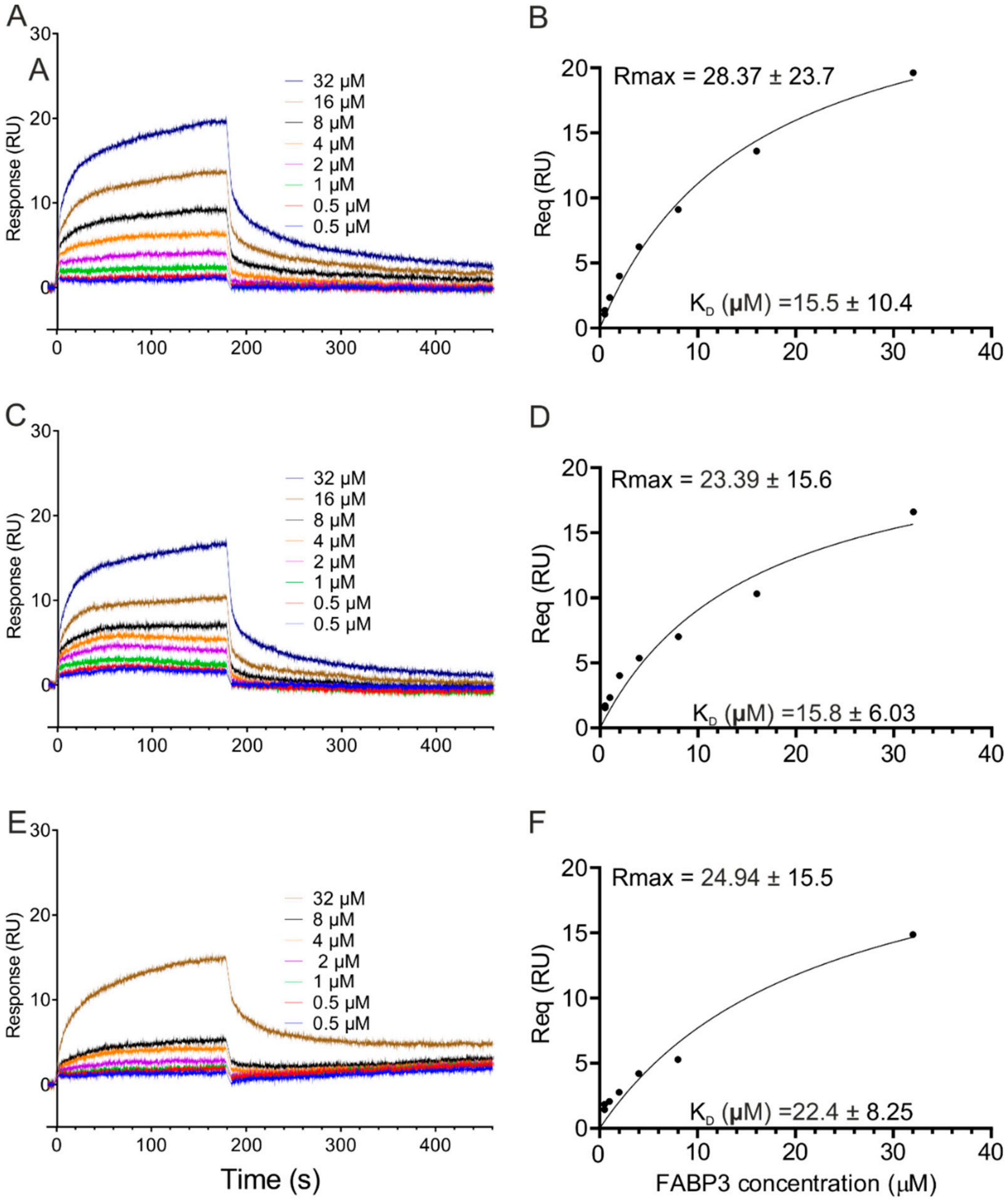


**Figure S1.** Measurement of the binding affinity between FABP3 and CooP peptide by using the surface plasmon resonance (SPR). (**A,B,E)** SPR sensorgrams and the steady state affinity between FABP3 and CooP peptide (**B,C,F)** from three independent experiments.

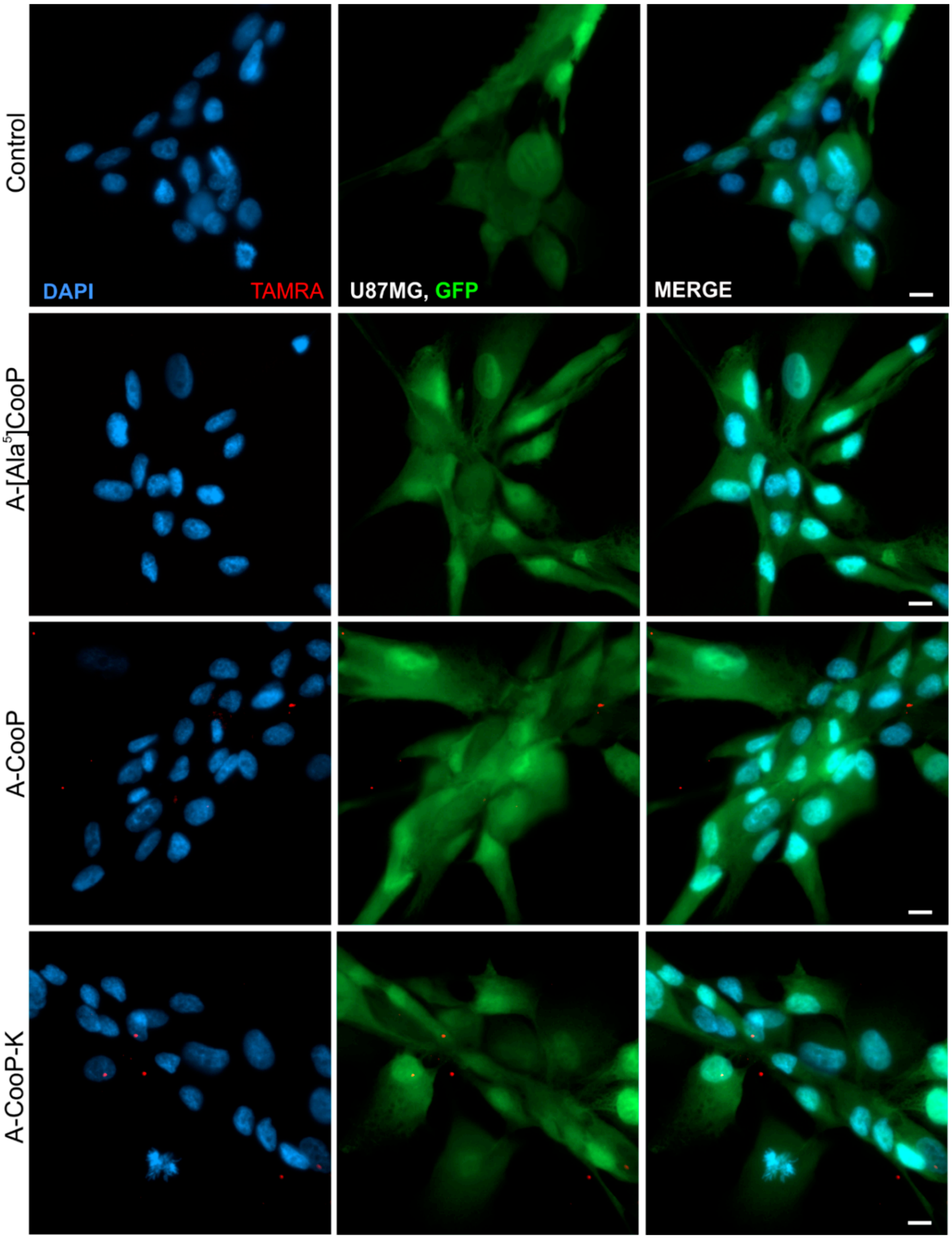


**Figure S2.** In vitro cellular uptake of the selected A-CooP variants by the U87MG cells. Microscopic images of the U87MG glioblastoma cells expressing GFP (green). These cells express only negligible amounts of FABP3. No significant binding or internalization of the control, A-[Ala$^5$]CooP, A-CooP, or A-CooP-K peptides (red) were observed.

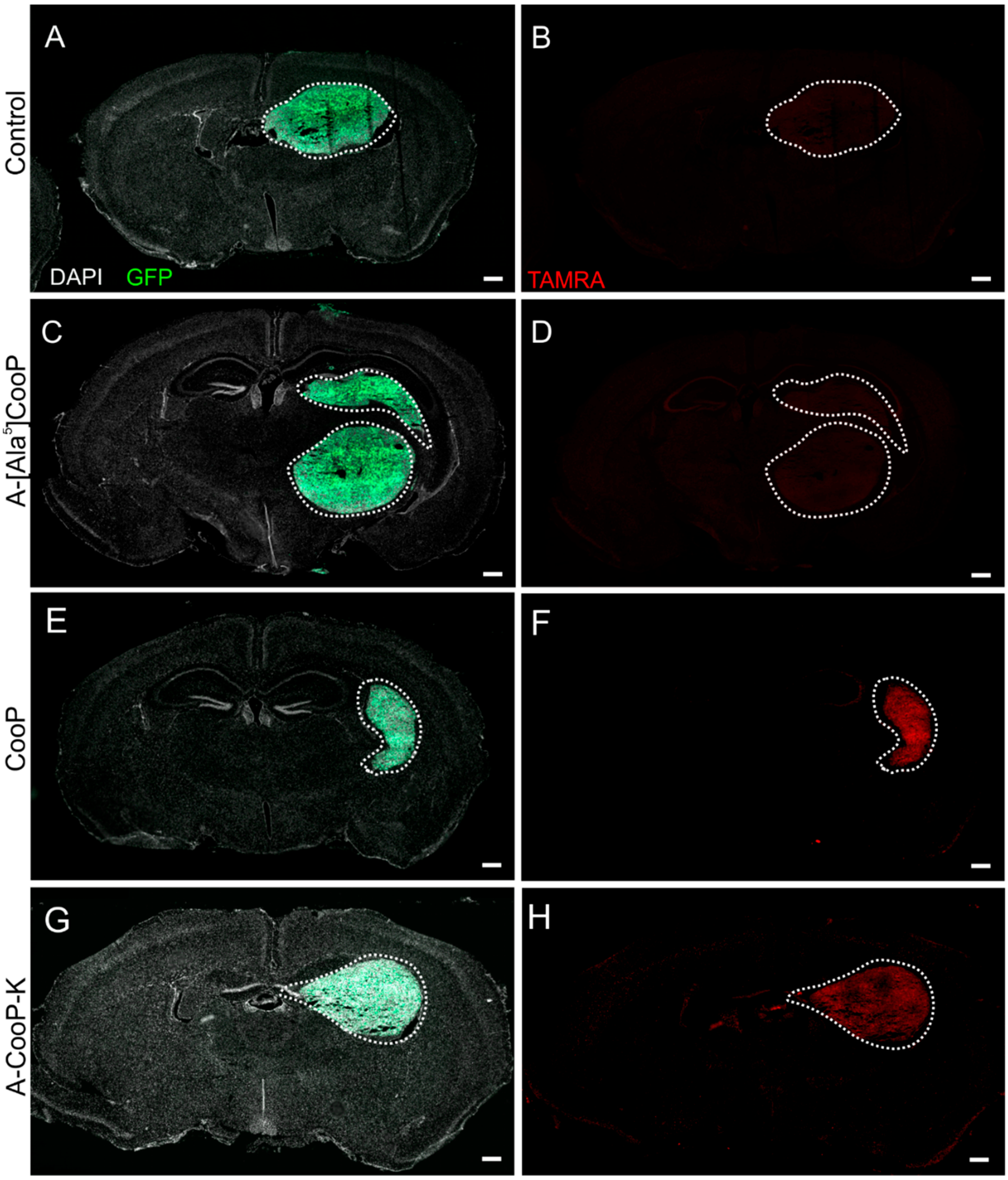


**Figure S3.** Homing of the A-CooP variants to the intracranial glioblastoma xenografts. The GFP-labeled patient-derived glioblastoma cells were intracranially grafted into immunocompromised mice. After 25 days fluorescently labeled peptides (100µM in 100 µl of 0.9% saline solution) were infused intravenously into tumor-bearing animals and let to circulate for 60 min. The brain tissue was collected and processed for immunofluorescence. Micrograghs show representative whole coronal section micrographs of murine brain containing the tumors (outlined with white dashed line) and the peptide-associated fluorescence in red. **(A-B) C**ontrol peptide, **(C-D)** A-[Ala$^5$]CooP, **(E-F)** A-CooP, and **(G-H)** A-CooP-K.

**Table S1.** MST summary table shows the calculated $K_D$ values, response amplitude (RU), standard error of regression, signal-to-noise ratio, and the reduced $X^2$ values ($R^2$) for the different peptide variants.

| **Peptide variants** | **Target conc. (nM)** | **$K_D$ (µM)** | **Response amplitude** | **Unbound** | **Bound** | **Standard error** | **$R^2$** | **Signal-to-noise** |
|---|---|---|---|---|---|---|---|---|
| A-CooP | 20 | 2.18 ± 1.15 | 15 | 842 | 827 | 2.43 | 0.60 | 7 |
| A-[$Ala^1$]CooP | 20 | - | - | - | - | - | - | - |
| A-[$Ala^2$]CooP | 20 | 0.09 ± 0.07 | 20 | 850 | 831 | 2.31 | 0.73 | 9 |
| A-[$Ala^3$]CooP | 20 | 0.25 ± 0.14 | 23 | 856 | 833 | 2.18 | 0.83 | 11 |
| A-[$Ala^4$]CooP | 20 | 0.11 ± 0.02 | 24 | 857 | 833 | 1.98 | 0.80 | 13 |
| A-[$Ala^5$]CooP | 20 | - | - | - | - | - | - | - |
| A-[$Ala^6$]CooP | 20 | 0.19 ± 0.03 | 14 | 845 | 831 | 1.34 | 0.73 | 11 |
| A-[$Ala^7$]CooP | 20 | 133 ± 75 | 138 | 839 | 977 | 6.37 | 0.73 | 23 |
| A-[$Ala^8$]CooP | 20 | 0.12 ± 0.05 | 24 | 851 | 828 | 2.34 | 0.84 | 11 |
| KA-CooP | 20 | 0.63 ± 0.13 | 15 | 843 | 828 | 1.72 | 0.76 | 10 |
| A-CooP-K | 20 | 0.07 ± 0.01 | 20 | 847 | 827 | 1.71 | 0.95 | 12 |
| RI-A-CooP | 20 | 24 ± 13 | 16 | 842 | 826 | 2.84 | 0.73 | 6 |
| Negative control | 20 | - | - | - | - | - | - | - |
