# Supplementary figures and images for: Tumor-Targeting Peptides: The Functional Screen of Glioblastoma Homing Peptides to the Target Protein FABP3 (MDGI)
Source: Cancers (Basel). 2020 Jul 8;12(7):1836. doi: 10.3390/cancers12071836 (PMC7409020; doi:10.3390/cancers12071836)

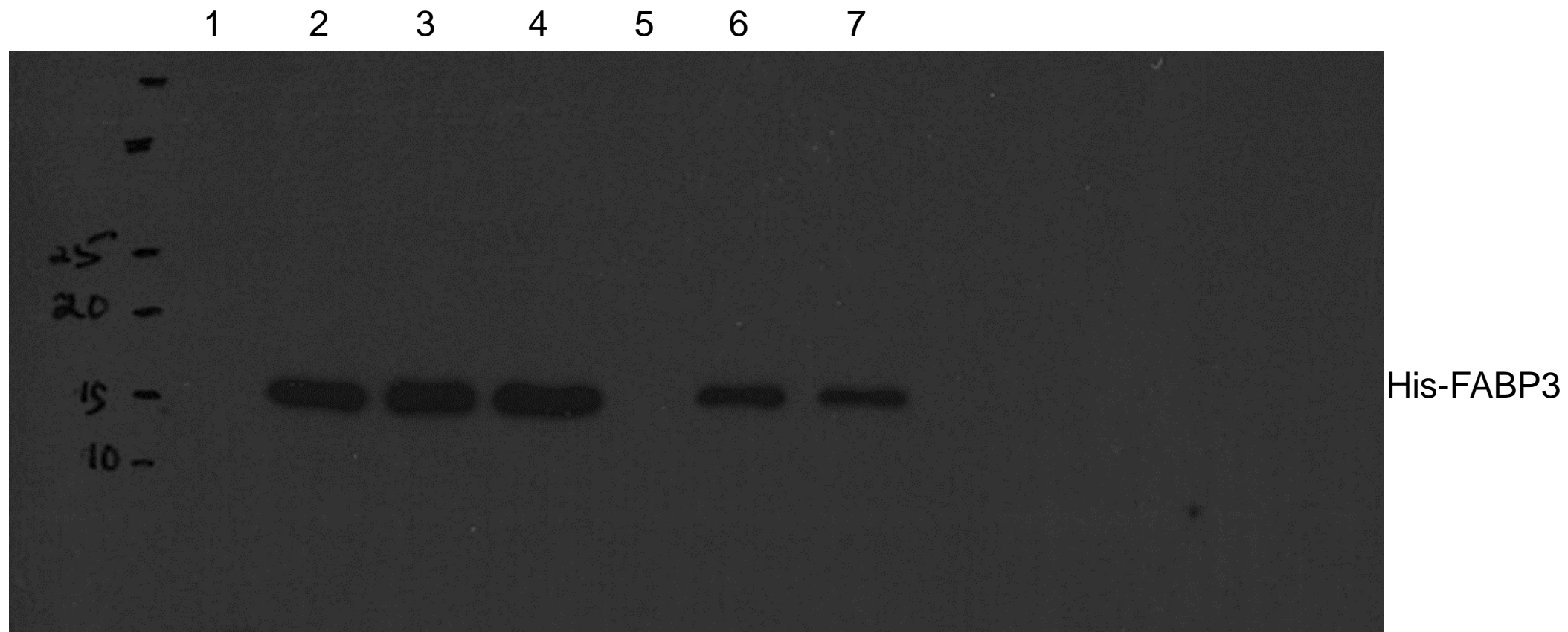

Figure 1A

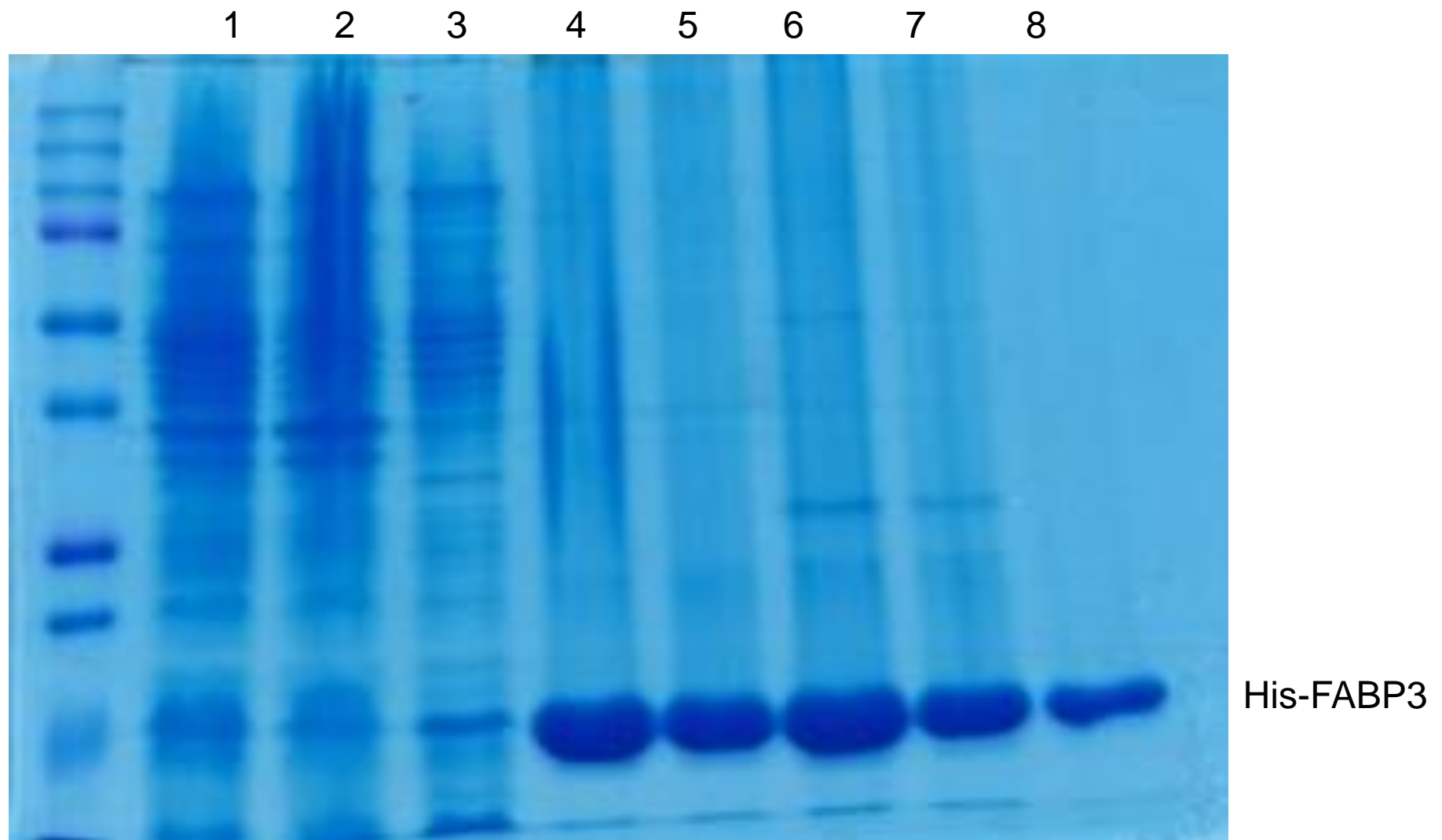

Figure 1B

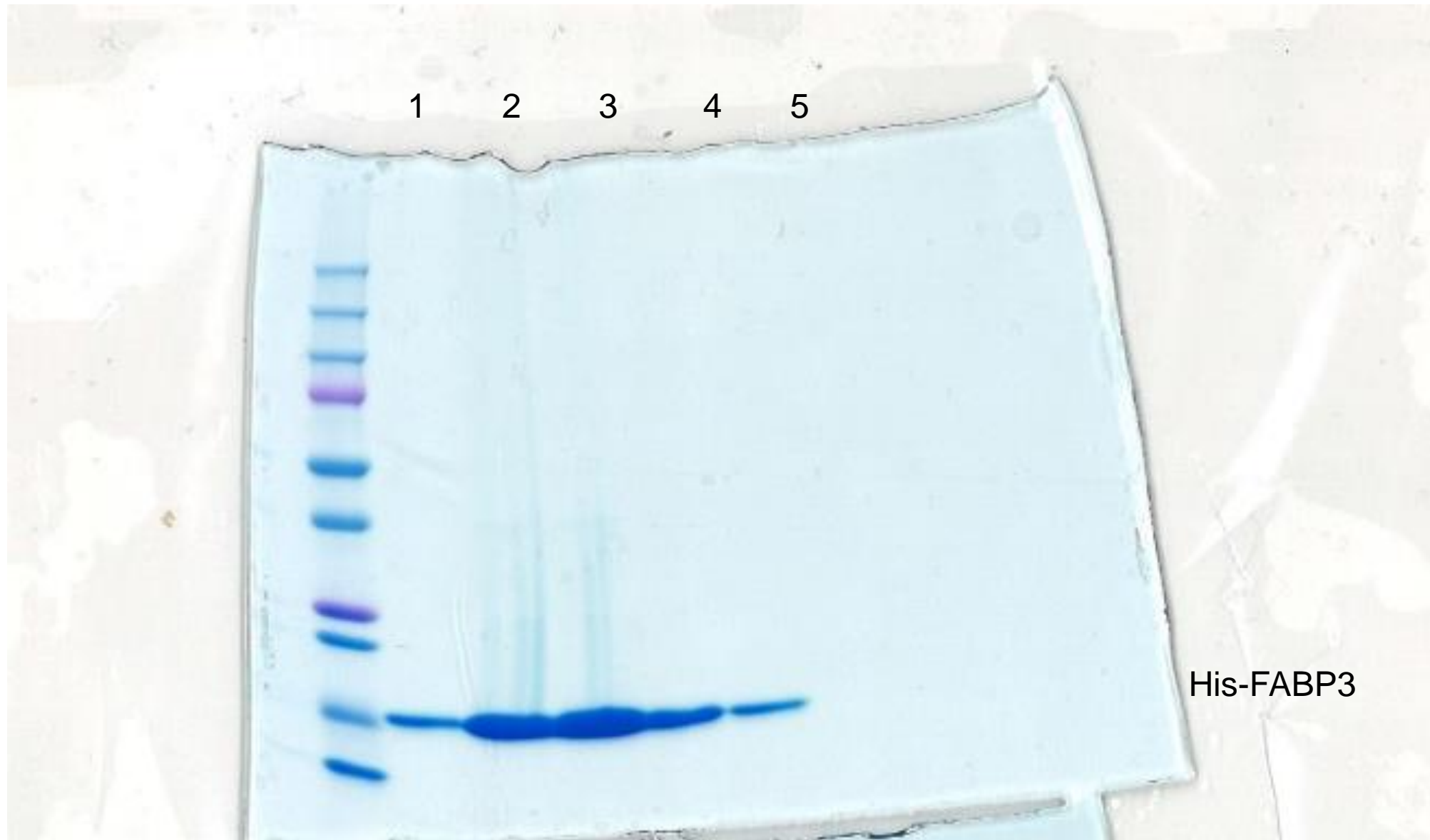

Figure 1C

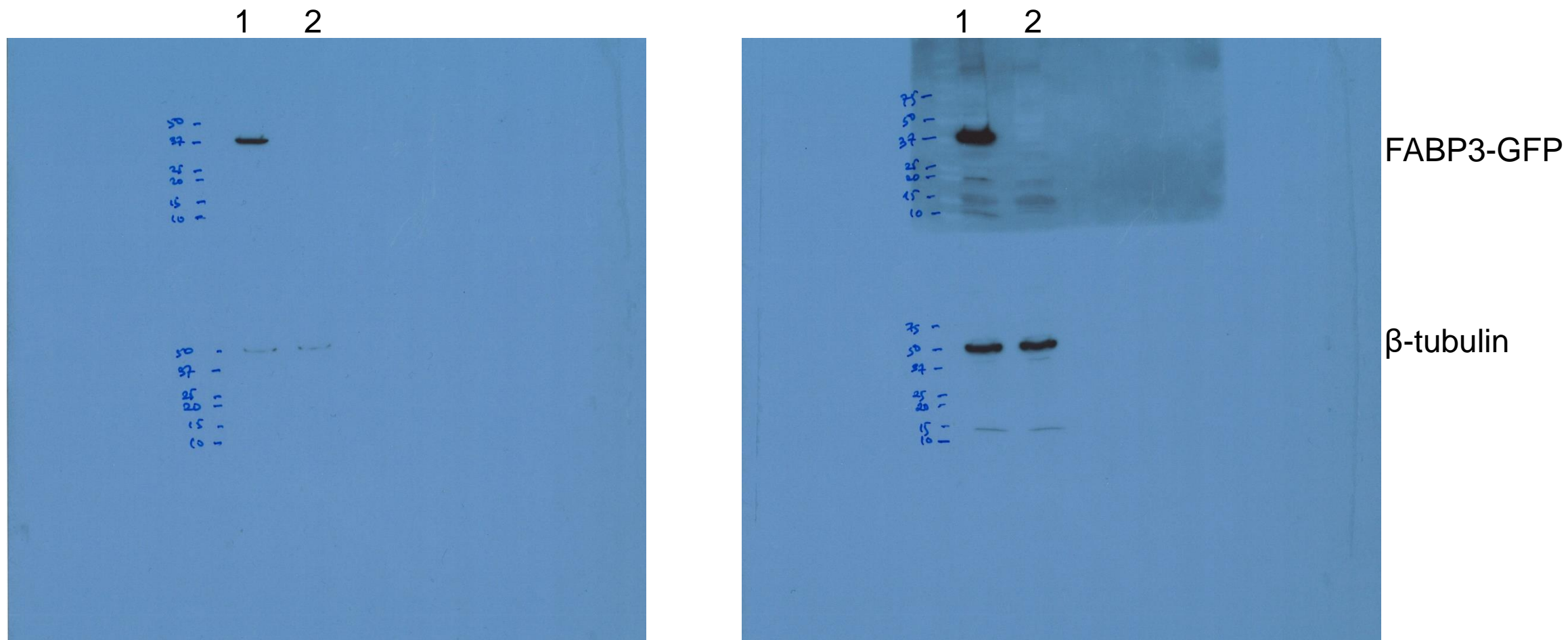

Figure 6A

Supplement: Supplementary file 1 [file cancers-12-01836-s001.zip › cancers-835234-original_images.pdf]
